# Non‐canonical IKB kinases regulate YAP/TAZ and pathological vascular remodeling behaviors in pulmonary artery smooth muscle cells

**DOI:** 10.14814/phy2.15999

**Published:** 2024-04-12

**Authors:** Aja Aravamudhan, Paul B. Dieffenbach, Kyoung Moo Choi, Patrick A. Link, Jeffrey A. Meridew, Andrew J. Haak, Laura E. Fredenburgh, Daniel J. Tschumperlin

**Affiliations:** ^1^ Department of Physiology and Biomedical Engineering Mayo Clinic Rochester Minnesota USA; ^2^ Division of Pulmonary and Critical Care Medicine, Department of Medicine Brigham and Women's Hospital Boston Massachusetts USA

**Keywords:** non‐canonical IKB kinases, pulmonary arterial hypertension, pulmonary artery smooth muscle cells, YAP and TAZ

## Abstract

Pulmonary arterial hypertension (PAH) causes pulmonary vascular remodeling, increasing pulmonary vascular resistance (PVR) and leading to right heart failure and death. Matrix stiffening early in the disease promotes remodeling in pulmonary artery smooth muscle cells (PASMCs), contributing to PAH pathogenesis. Our research identified YAP and TAZ as key drivers of the mechanobiological feedback loop in PASMCs, suggesting targeting them could mitigate remodeling. However, YAP/TAZ are ubiquitously expressed and carry out diverse functions, necessitating a cell‐specific approach. Our previous work demonstrated that targeting non‐canonical IKB kinase TBK1 reduced YAP/TAZ activation in human lung fibroblasts. Here, we investigate non‐canonical IKB kinases TBK1 and IKKε in pulmonary hypertension (PH) and their potential to modulate PASMC pathogenic remodeling by regulating YAP/TAZ. We show that TBK1 and IKKε are activated in PASMCs in a rat PH model. Inflammatory cytokines, elevated in PAH, activate these kinases in human PASMCs. Inhibiting TBK1/IKKε expression/activity significantly reduces PAH‐associated PASMC remodeling, with longer‐lasting effects on YAP/TAZ than treprostinil, an approved PAH therapy. These results show that non‐canonical IKB kinases regulate YAP/TAZ in PASMCs and may offer a novel approach for reducing vascular remodeling in PAH.

## 1 INTRODUCTION

Pulmonary arterial hypertension (PAH) is characterized by pulmonary vascular remodeling, increased pulmonary vascular resistance (PVR), progressive pulmonary arterial (PA) stiffening (Malenfant et al., 2013), and ultimately right ventricular (RV) heart failure and death. Pulmonary artery smooth muscle cells (PASMCs) are one of the key cell types contributing to pathological vascular remodeling in PAH. While the biological mechanisms underlying PAH pathogenesis are incompletely understood, PA stiffening initiates a pathological feedback loop controlled by the mechanoregulatory transcription factors YAP/TAZ (Xie et al., 2018; Zuo et al., 2021). Pharmacological targeting of YAP/TAZ may thus provide a novel means to attenuate and/or reverse PAH and prevent RV dysfunction (Bertero et al., 2015; Pullamsetti et al., 2017). The prostacyclin pathway is one of the key targets of currently approved FDA treatments in PAH (Sitbon & Noordegraaf, 2017). Interestingly, recent work suggests that one of the mechanisms of action of prostacyclin analogs and prostacyclin receptor agonists may be via inhibition of YAP/TAZ (Dieffenbach et al., 2017; Zmajkovicova et al., 2019). However, due to the short half‐life of prostacyclin, prostanoid therapy requires intravenous administration or frequent dosing, commonly leading to side effects, and may lead to complications such as catheter‐related infections in patients requiring continuous infusion (Fares, 2015; Galie et al., 2009). These limitations of prostanoid therapy highlight the importance of developing new therapeutics that are both highly effective and long acting for the treatment of PAH.

TANK (TRAF‐associated NF‐κB activator) binding kinase 1 (TBK1) and IKKε (also known as IKK‐inducible or IKK‐i) are termed the non‐canonical IKB kinases (Peters et al., 2000; Pomerantz & Baltimore, 1999; Shimada et al., 1999). Prior studies have shown that TBK1/IKKε can interact with YAP/TAZ leading to inhibition of TBK1/IKKε activation during the host antiviral cellular response (Garcia et al., 2020; Zhang et al., 2017). We recently demonstrated that TBK1 can regulate TGF‐β signaling and the stability of transcription co‐factors YAP/TAZ in human pulmonary fibroblasts (Aravamudhan et al., 2020), controlling their fibrogenic phenotype. Hence, the interaction between TBK1/IKKε and YAP/TAZ is diverse and cell‐type specific. In PAH, non‐canonical IKB kinase pathway associated gene activation has been observed in genomic studies in pulmonary hypertension samples (Stearman et al., 2019) and in a meta‐analysis of blood genome‐wide expression profiling studies in PAH (Elinoff et al., 2020), suggesting potential relevance to PAH pathogenesis.

PAH progression is known to be driven by a multitude of interacting pathways. Mutation of the TGF‐β family member BMPRII has been observed in familial PAH (~27% of cases) (Aldred et al., 2006; Cogan et al., 2006; Newman et al., 2004), and dysregulation of BMPR2 signaling has also been implicated in the pathogenesis of idiopathic PAH (Andruska & Spiekerkoetter, 2018). Numerous studies suggest that inflammation plays a key role in the development of pulmonary hypertension (PH) with strong evidence from animal models and human patient samples that exhibit inflammatory foci (Dorfmüller et al., 2003; Rohm et al., 2019). Furthermore, inflammatory mediators that are known to activate non‐canonical IKB kinases (Brasier, 2010; Patel et al., 2009; Yu et al., 2012) have been shown to be prognostic factors for worse outcomes in PAH (Dorfmüller et al., 2003). Further research has shown that the lack of protective effects from BMPRII signaling along with increased inflammatory activity (facilitated by cytokines like IL‐6) may be a major factor promoting vascular remodeling of smooth muscle and endothelial cells of the pulmonary vasculature in PAH (Hagen et al., 2007; Hiepen et al., 2019; Steiner et al., 2009). The links between inflammatory and remodeling events in PAH remain to be fully elucidated.

We have shown previously that a mechanobiological feedback loop fueled by YAP/TAZ contributes to the sustained vascular remodeling pathology in PAH (Dieffenbach et al., 2017). While inflammatory cytokines such as IL‐6 and transcriptional regulators YAP/TAZ play a pivotal role in PAH pathogenesis, they play several critical roles in cellular defense and regeneration and therefore cannot be directly targeted without undesirable effects. Studies on signaling by non‐canonical IKB kinases have shown that their signaling is highly cell‐type specific and dependent on the nature of the activating signal and the signaling subcomplex present in a given cell (Aravamudhan et al., 2020; Elinoff et al., 2020; Garcia et al., 2020; Stearman et al., 2019; Zhang et al., 2017). This renders the non‐canonical IKB kinases as a complex signaling hub capable of differentially controlling several cellular processes and making them an attractive therapeutic target in diseases such as PAH. The continued discovery of chemical inhibitors of these kinases also offers a diverse toolkit to clinically modulate them (Aldred et al., 2006). Here, we tested if inhibition of TBK1/IKKε would reduce YAP/TAZ activation and attenuate pathological vascular remodeling behaviors in human PASMCs.

## 2 METHODS

### 2.1 Animal models of PH

All animal experiments were performed in compliance with the relevant laws and guidelines as set forth by the Harvard Medical Area Standing Committee on Animals and the Lovelace Respiratory Research Institute Animal Care and Use Committee (IACUC) under IACUC‐approved protocols. Adult male Sprague‐Dawley rats (Charles River Laboratories) were injected with a single dose of monocrotaline (MCT) (50 mg/kg) or vehicle (PBS) subcutaneously (Landt et al., 2012). Animals were euthanized at 4 week, at which time lungs were snap‐frozen in liquid nitrogen and stored at −80°C. The primary results from these animals were previously reported (Liu et al., 2016), including significant elevation in right ventricular systolic pressure, Fulton's index, and arterial wall thickness in response to MCT.

### 2.2 Immunohistochemistry

The snap‐frozen lungs were immersed in 20% sucrose solution, embedded in OCT, and sectioned as 10 μm slices for histological staining, examination, and quantification. The rat lung tissue 10 μm sections from control (vehicle treated) and MCT‐treated rats were fixed in 4% PFA and stained for active (phosphorylated) TBK1 and IKKε along with α‐SMA to mark the vascular smooth muscle cells in the pulmonary vasculature. The sections were blocked with 10% normal donkey serum/1% BSA/0.3 M Glycine/0.5% Triton X100 in 1X TBS for 60 min. The sections were stained with primary antibody (Rabbit (DA1E) mAb IgG XP® Isotype Control, 1:100, 3900, Cell signaling technology; or rabbit anti‐pTBK1(Ser172) 1:100, Bios‐bs‐3400R, Bioss USA; or rabbit anti‐pIKKε(Ser172) 1:100, 06–1340, Millipore Sigma; along with goat anti‐α‐SMA 1:200, NB300‐978, Novus biologics) diluted in antibody buffer (1% BSA, 0.5% Triton X100 in 1X TBS) and incubated at 4°C overnight, washed 3X with 1x TBST (0.25% Triton X100 in 1X TBS), followed by incubation with fluorescent dye conjugated secondary antibody (Alexa Fluor 555 Donkey anti‐rabbit Ig‐G 1:1000, A31572, Life Technologies; and Alexa Fluor 488 donkey anti‐goat IgG 1:1000, A11055, Life Technologies) and DAPI (1:1000, 62248, Thermo scientific) in antibody buffer for 60 min at room temperature. Stained sections were washed 4x with 1x TBST, mounted in Aqua‐Poly/Mount (Polyscience Inc., 18606) and cover slipped. The slides were dried overnight, sealed with clear nail polish, and imaged with a Keyence microscope at 20X, and 40X magnifications. The 40X images were used for quantification of active TBK1 and IKKε in the smooth muscle cells of the pulmonary vasculature (identified by α‐SMA staining).

### 2.3 Quantification of pTBK1 and pIKKε staining in pulmonary vascular smooth muscle cells

Immunofluorescent staining of pTBK1 and pIKKε were analyzed in the α‐SMA positive pulmonary vascular wall cells. Thresholding followed by pixel intensity and area quantification were performed using ImageJ. The pixel intensity of the stain was normalized to the section's area. All quantification was performed on 10 or more randomly chosen images per sample (Aravamudhan et al., 2018; Ruffenach et al., 2019). *N* = 4 samples/group were used for control and MCT samples.

### 2.4 Cell culture

Primary Pulmonary Artery Smooth Muscle Cells (PASMCs) (Human PASMCs, lot no. 0000669096 and lot no. 0000701036) were purchased from Lonza (Walkersville, MD). 0000669096 and 0000701036 were derived from 51‐year‐old and 58‐year‐old Male Caucasian patients, respectively. Control and PAH PASMCs were derived from explanted lungs of patients with group 1 PAH who underwent lung transplantation or from control donor lungs not suitable for transplantation as part of the Pulmonary Hypertension Breakthrough Initiative (PHBI) under a protocol approved by the Partners Human Research Committee. Informed consent was obtained by the PHBI subjects or their legal guardians before they enrolled in the study. Primary idiopathic pulmonary arterial hypertension (IPAH) PASMCs and control PASMCs were studied as in prior work (Dieffenbach et al., 2017). IPAH Donor 1 and matched Control Donor 1 PASMCs were from 32‐ and 36‐year‐old female subjects, respectively. IPAH Donor 2 and matched control Donor 2 PASMCs were from 25‐year‐old male patients, respectively. Cell lines tested negative for human immunodeficiency virus, hepatitis B, hepatitis C, mycoplasma, bacteria, yeast, and fungi. PASMCs were cultured in smooth muscle basal medium (SmBM) supplemented with 5% FBS, SmGM‐2 SingleQuots (Lonza), penicillin (100 IU/m), and streptomycin (100 μg/mL) in a humidified incubator (21% O2, 5% CO2) at 37°C. All primary cell culture experiments were performed with cells at passage six or less.

### 2.5 Chemical inhibitors

The following chemical inhibitors were used: treprostinil (Compound CID: 91617675) a potent prostacyclin (PGI2) analog (Tocris), inhibitors of TBK1/IKKε: Amlexanox (Compound CID: 2161) (Tocris), TBK1/IKKε‐IN‐1(compound I) (CI) (Compound CID: 124156234) (Selleckchem), and TBK1/IKKε‐IN‐2 (compound II) (CII) (Selleckchem). The chemical stocks were prepared using DMSO as the solvent and all the experiments used DMSO as a control.

### 2.6 Cytokine treatment

PASMCs were seeded at a density of 25 cell/mm^2^ in 24‐well plates and cultured at 37°C and 5% CO_2_. DMEM/F12 media was used with 5% FBS. Following cellular attachment, the cells were serum starved (SS) in DMEM/F12 with 0.5% FBS, penicillin (100 IU/m), and streptomycin (100 μg/mL) overnight. PASMCs were subsequently treated with SS media, high serum (HS) media (DMEM/F12 and 20.0% FBS, penicillin (100 IU/m), and streptomycin (100 μg/mL)), TNF‐α (20 ng/mL), IL‐1β (10 ng/mL), or ET‐1 (100 ng/mL). DMEM/F12 and HS (20.0% FBS, penicillin (100 IU/m), and streptomycin (100 μg/mL)) media was used as the positive control. The cells were lysed, and the contents collected after 2 h of treatment. For YAP/TAZ staining, the same protocol was followed while the cells were seeded in a 96 well plate and fixed after 2 h for immunostaining.

### 2.7 TBK1/IKKε plasmid transfection

pcDNA3‐Flag‐TBK1 and pcDNA3‐Flag‐TBK1(K38A) were generously donated by Dr. Fitzgerald (UMass Medical, Boston) (Fitzgerald et al., 2003). pcDNA3 IKKe Flag, pcDNA3 IKKe K38A Flag (Fitzgerald et al., 2003), and Flag pcDNA3 (Sanjabi et al., 2005) were purchased from Addgene. Briefly, the plasmids were transformed into competent cells after recovery, as per the manufacturer's instructions. LB agar plates with appropriate antibiotics were used to select the colonies by incubating at 37°C overnight. These selected colonies expressing the plasmids of interest were further scaled up and cultured in sterile LB broth with Carbenicillin. After incubation for 18 h, the culture was spun down in a centrifuge to isolate the bacterial plasmids. Plasmids were purified using a Maxiprep kit (Qiagen). The purified plasmids were dissolved in TE buffer and spectrophotometrically quantified. PASMCs were seeded in 6‐well plates and allowed to reach 80% confluence. Lipofectamine 3000 reagent (Thermo Fisher Scientific) was used to transfect 2.5 μg of plasmid per reaction.

### 2.8 Western blot analysis

PASMCs were plated in six‐well plates. For studies measuring phosphorylated and total TBK1/IKKε in cells treated with cytokines/chemicals, the cells were plated, and serum starved overnight, and then, media with cytokine/chemicals was added to the media for 2 h before protein isolation and blotting for TBK1/IKKε activation. A seeding density of 50 cells/mm^2^ was used in all experiments. In all experiments, the total protein was isolated using RIPA buffer (pH 8.0) with Pierce Phosphatase Inhibitor (Thermo) and Halt Protease Inhibitor Cocktail (Thermo). Total protein concentration of the lysates was determined using a Pierce BCA Protein Assay Kit (Thermo), and samples were run on a 7.5% polyacrylamide gel. Proteins were then transferred from the gel to a nitrocellulose membrane. The following primary antibodies were used for overnight incubation of these blots: anti‐phospho‐TBK1/NAK (Ser172) (D52C2) XP Rabbit mAb (5483 T; Cell Signaling Technology), Anti‐phospho‐IKK‐epsilon (Ser172) (06–1304; Millipore‐Sigma), Anti‐NAK/TBK1 antibody (EP611Y) (ab40676; Abcam, Cambridge, MA), Anti‐IKKε (D20G4) Rabbit mAb (2905S; Cell Signaling Technology), Anti‐YAP/TAZ rabbit mAb (8418S; Cell Signaling Technology), and GAPDH (14C10; Cell Signaling Technology). All antibodies were diluted in 5% BSA/TBST. Phospho‐TBK1 was diluted at 1:250, and phospho‐IKKε and IKKε were diluted at 1:500 and TBK1 at 1:1000. Blots were washed with TBS‐Tween before 60 min incubation with anti‐rabbit‐HRP‐conjugated secondary (W4011; Promega) antibody diluted 1:3000 in 5% BSA/TBST. Membranes were imaged using a Bio‐Rad ChemiDoc Imaging system (Hercules, CA) with quantification performed via densitometry using NIH‐Fiji software. Each antibody produced one obvious band of the appropriate size, and TBK1 and IKKε antibodies were validated using TBK1 and IKBKE siRNA.

### 2.9 Immunostaining

PASMCs were plated in clear‐bottom 96‐well plates at a density of 50 cells/mm^2^ in DMEM/F12 (Gibco) containing SS media overnight as described above. In experiments testing CII, the different concentrations of the drug were diluted in cell culture media before cell treatment. In siRNA experiments, PASMCs were first transfected with control or (TBK1+IKKε)‐siRNA for 24 h, reseeded on a 96‐well plate, SS media for 48 h. Cells were fixed in 3.7% formalin (Sigma), permeabilized with 0.25% Triton X‐100 (Sigma‐Aldrich), and then blocked with 5% BSA for 1 h. Cells were incubated overnight with a FITC‐conjugated mouse monoclonal antibody against YAP/TAZ (D24E4; Cell Signaling Technology) diluted 1:200 in PBS with 1% BSA. Finally, the cells were counterstained with DAPI nuclear stain. Cells were then washed and imaged using a Cytation 5 imaging system (Bio‐Tek) at 10 X magnification. YAP/TAZ localization was quantified using Gen5 (Biotek) software. For this purpose, cell nuclei were identified using the DAPI channel and YAP/TAZ nuclear staining intensity was first quantified in sparsely seeded PASMCs as a positive control. A threshold value of 90% of this intensity was then set to quantify the cells positive for nuclear YAP/TAZ. The percentage of YAP/TAZ nuclear positive cells after treatments was thus calculated (Haak et al., 2019).

### 2.10 SiRNA transfection

Control, IPAH subject, and Lonza PASMCs were transfected using Lipofectamine RNAiMAX (Life Technologies) with an siRNA (Dharmacon, Lafayette, CO) containing four siGENOME Human TBK1 (29110) and siGENOME Human IKBKE (9641) siRNA or a nontargeting SMARTpool control. Cells were seeded at a density of 100 cells/mm^2^ in 24‐well plates using SS media, as described above, and treated with 10 nM of each targeting siRNA SMARTpool or equivalent amounts of nontargeting siRNA for at least 16 h, with a reference sample collected at the time of siRNA treatment to serve as a baseline control. Knockdown of each siRNA target was confirmed by RT‐PCR and western blotting 72 h after transfection. Gene expression analysis was performed by qPCR to confirm knockdown. Cells received different treatments as indicated in each experiment. RNA was isolated using the RNeasy Plus Mini Kit (Qiagen, Germantown, MD) as per the manufacturer's protocol. Isolated RNA was used to synthesize cDNA using SuperScript VILO (Invitrogen Life Technologies). Fast Start Essential DNA Green Master (Roche) was used to perform quantitative PCR analysis using a LightCycler 96 (Roche) machine. ΔΔCt calculation was used relative to 16srRNA (16 s) to determine the fold change in gene expression of the samples with respect to the nontreated controls.

### 2.11 Cell proliferation

Proliferation was assessed using the BrdU Cell Proliferation colorimetric ELISA Kit (Abcam, Boston, MA) as per the manufacturer's protocol. PASMCs were seeded using a sterile 96‐well tissue culture plate at a density of 2 × 10^5^ cells/mL in 100 μL/well of appropriate cell culture media. Some of the wells on the plate were used for controls: wells that do not contain cells (media alone) and wells which contain cells but did not receive the BrdU reagent (assay background). In total, 20 μL of the diluted 1X BrdU reagent was added to each well 24 h before harvest for every timepoint. At set timepoints, the plates were harvested by adding 200 μL/well of the fixing solution and incubated at room temperature for 30 min to fix the cells and denature the DNA to be assayed for BrdU incorporation. The plates were washed and incubated with 100 μL/well of anti‐BrdU monoclonal Detector Antibody for 1 h at room temperature. The plates were washed and incubated with 100 μL/well of 1X Peroxidase Goat Anti‐Mouse IgG Conjugate for 30 min. Plates were washed with distilled water and dried off by patting, and 100 μL/well of TMB Peroxidase substrate was added and incubated for 30 min at room temperature in the dark. The plates were read on a spectrophotometric plate reader at 450/550 nm. A standard curve with known numbers of cells was used to calculate the proliferation percentages.

### 2.12 Cell viability and apoptosis

Cellular viability and apoptosis were measured using an Apo‐Glo® Live multiplex assay (Promega, Madison, WI) as per the manufacturer's protocol. Briefly, the cells were plated as above on a white assay plate specifically used for the Apo‐Glo Live assay. At the end of each timepoint, 10 μL of substrate was transferred into 2 mL of Assay Buffer. In total, 20 μL of viability reagent was added to all wells, and briefly mixed by orbital shaking (300–500 rpm for ~30 s). The cells were incubated for 30 min at 37°C. Fluorescence was measured at the following wavelength: 400Ex/505Em. Fluorescence was used as directly proportional to cell viability (RFU).

Apoptosis: Caspase‐Glo® 3/7 Buffer and lyophilized Caspase‐Glo® 3/7 Substrate were equilibrated to room temperature before use. The contents of the Caspase‐Glo® 3/7 Buffer bottle were transferred into the amber bottle containing the Caspase‐Glo® 3/7 Substrate. The contents were mixed until the substrate was thoroughly dissolved to form the Caspase‐Glo® 3/7 Reagent. In total, 100 μL of Caspase‐Glo® 3/7 Reagent was added to each sample in the 96‐well plate containing 100 μL of media. Samples were mixed gently using a plate shaker and incubated at room temperature for 30 min. The luminescence of the samples was measured in a plate‐reading luminometer as caspase (apoptotic) activity and normalized to cellular viability.

### 2.13 Cell numbers

PASMC cell number was quantified using a CyQUANT® assay (Thermo Fisher scientific Waltham, MA) as per the manufacturer's protocol. Briefly, PASMCs were seeded in 96‐well plates as above. The microplates were emptied by gentle inversion and blotted onto paper towels to remove medium from the wells. The wells were washed carefully with phosphate buffered saline (PBS), frozen and stored at −70°C until samples were assayed. The concentrated cell‐lysis buffer stock solution (Component B) was diluted 20‐fold in distilled water. The CyQUANT® GR stock solution (Component A) was diluted 400‐fold in the 1X cell‐lysis buffer. The plates were thawed at room temperature. Then, 200 μL of the CyQUANT® GR dye/cell‐lysis buffer was added to each sample well. It was mixed gently and incubated for 2–5 min at room temperature, protected from light. The sample fluorescence was measured using a fluorescence microplate reader with filters appropriate for ~480 nm excitation and ~520 nm emission maxima. A standard curve was used to determine the cell number from the fluorescence values obtained in the assay.

### 2.14 Gene expression analysis by qPCR

Cells received different treatments as indicated in each experiment before RNA isolation using the RNeasy Plus Mini Kit (Qiagen) as per the manufacturer's protocol. Isolated RNA was used to synthesize cDNA using SuperScript VILO (Invitrogen Life Technologies). Fast Start Essential DNA Green Master (Roche) was used to perform quantitative PCR analysis using a LightCycler 96 (Roche) machine. ΔΔCt calculation was used relative to 16srRNA (16 s) to determine the fold change in gene expression of the samples with respect to the nontreated controls.

**Table:** 

Human‐YAP1‐F	TGTCCCAGATGAACGTCACAGC
Human‐YAP1‐R	TGGTGGCTGTTTCACTGGAGCA
Human‐WWTR1‐F	GAGGACTTCCTCAGCAATGTGG
Human‐WWTR1‐R	CGTTTGTTCCTGGAAGACAGTCA
Human‐CTGF‐F	GTCCAGCACGAGGCTCA
Human‐CTGF‐R	TCGCCTTCGTGGTCCTC
Human‐CYR61‐F	CTCGCCTTAGTCGTCACCC
Human‐CYR61‐R	CGCCGAAGTTGCATTCCAG
Human‐TBK1‐F	TGCACCCTGATATGTATGAGAGA
Human‐TBK1‐R	AAATGGCAGTGATCCAGTAGC
Human‐IKBKE‐F	GAGAAGTTCGTCTCGGTCTATGG
Human‐IKBKE‐R	TGCATGGTACAAGGTCACTCC
Human‐16srRNA‐F	GCTTTCCTTTTCCGGTTGCG
Human‐16srRNA‐R	ACACGGATGTCTACACCAGC

### 2.15 Traction force microscopy

As previously described (Marinković et al., 2012), polyacrylamide gel substrates with 13 kPa Young's moduli were prepared, and fluorescent sulfate‐modified latex microspheres (0.2 m, 505/515 excitation/emission, FluoSpheres; Life Technologies) were conjugated to the gel surfaces after treatment with 1 mg/mL of dopamine hydrochloride (Sigma‐Aldrich) in 50 mM HEPES solution (pH 8.5) for 20 min, followed by treatment with type 1 collagen solution (Advanced Biomatrix). Cells were plated on the gels and switched to DMEM/F12 containing 2% FBS overnight. For inhibitor experiments, the drug was added to the culture media with TGF‐□ (6 ng/mL) and ET‐1 (100 ng/mL). In siRNA experiments, PASMCs were first transfected with control or TBK1/IKBKE siRNA and then reseeded on gels after a 24‐h transfection period. Images of gel surface‐conjugated fluorescent beads were obtained for cells before and after trypsinization using a Cytation5 imaging system (Bio‐Tek) at 10X magnification. TractionsForAll (https://www.mayo.edu/research/labs/tissue‐repair‐mechanobiology/software) software was used to measure the two‐dimensional traction forces by measuring bead displacement fields and computing corresponding traction fields.

### 2.16 Statistical analysis

Data are shown as means +/− SD. Comparisons involving multiple groups were performed using one‐way ANOVA with Tukey post‐test. Comparisons involving subgroups were performed using a two‐way ANOVA with a multiple‐comparison post‐test. Comparisons involving two groups were performed using a t‐test (GraphPad Prism v 8.0; GraphPad Software, San Diego, CA). *p* < 0.05 was considered significant.

## 3 RESULTS

### 3.1 TBK1/IKKε are activated in the pulmonary vasculature of MCT‐induced PH in rats

**FIGURE 1:**
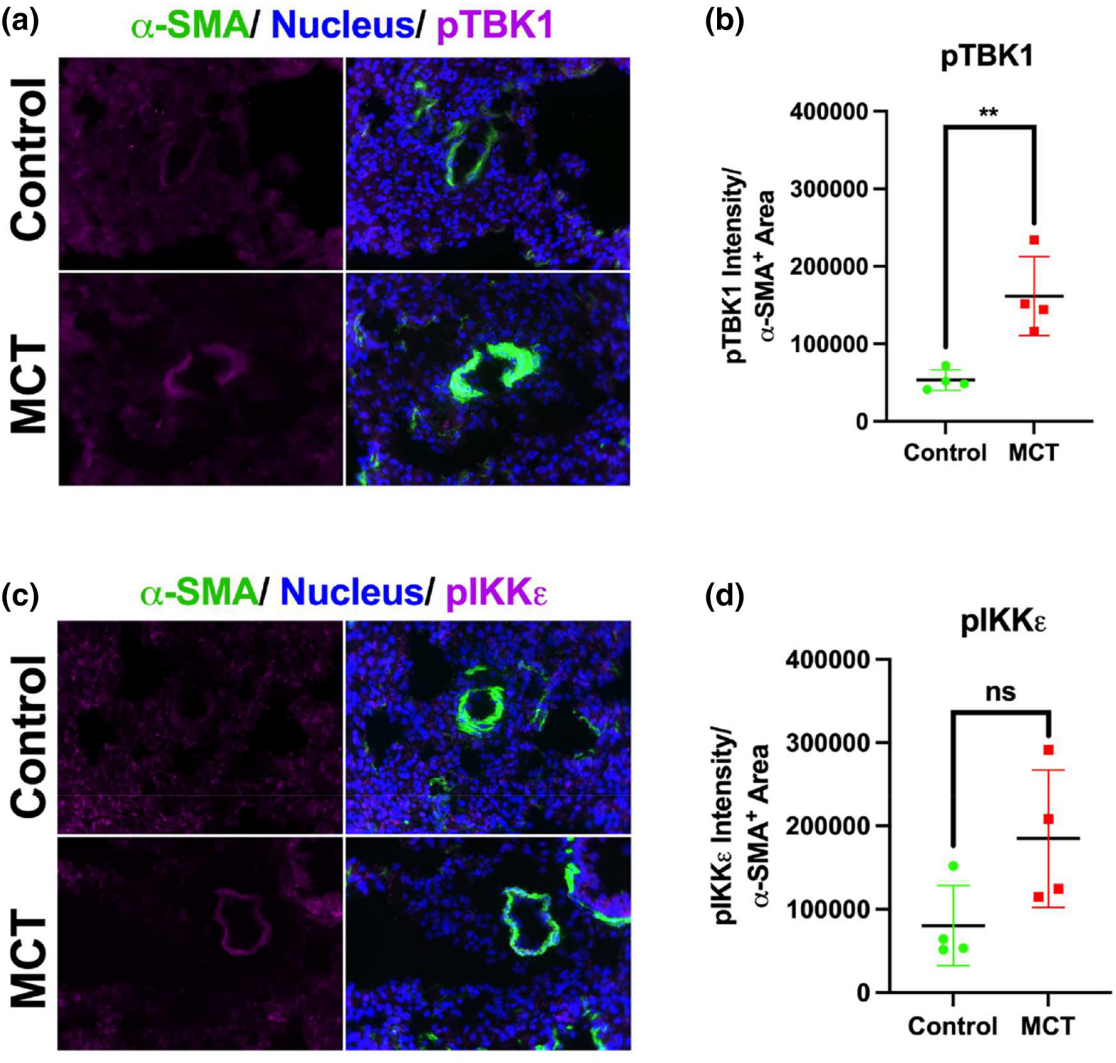
Activation of TBK1/IKKε in pulmonary vasculature of MCT rats. Histological sections from the lungs of Control (vehicle) treated vs monocrotaline (MCT) treated rats after 4 weeks of treatment were stained for (a) active pTBK1 (purple)/nucleus (DAPI) (blue) and smooth muscle cells (α‐SMA) (green) and (c) active pIKKε (purple)/nucleus (DAPI) (blue) and smooth muscle cells (α‐SMA) (green). A minimum of 10 vessels were captured per rate, and the pTBK1 (b) and pIKKε (d) protein fluorescent intensity within the smooth muscle (α‐SMA+ area) was measured for each vessel and averaged. Groups were compared by unpaired *t*‐test with ** indicates *p* value <0.01, Data represent four biological samples per condition. Scalebar represents 100 μm.

To examine if non‐canonical IKB kinases are activated in PASMCs in experimental PH, lungs from a previously published study (Liu et al., 2016) of monocrotaline (MCT)‐induced PH rats were histologically analyzed. Vehicle control and MCT‐treated rat lungs were stained using antibodies against activated TBK1 and IKKε, with αSMA co‐staining used to identify smooth muscle cells in the pulmonary vascular wall (green). Active TBK1 and IKKε were stained with pTBK1 (purple) (Figure 1a) and pIKKε antibodies (purple) (Figure 1c). The intensity of the signals in the αSMA‐expressing pulmonary vascular smooth muscle cells was quantified and normalized to the vessel wall area. Expression of active pTBK1 was significantly higher in MCT‐treated rats compared with vehicle‐treated controls (Figure 1b). There was a trend toward increased expression of pIKKε in MCT rats compared to controls (Figure 1d, *p* = 0.07), but expression was highly variable among animals. These results are consistent with increased activation of non‐canonical IKB kinases TBK1 and IKKε in PASMCs in experimental PH.

### 3.2 Cytokine stimulation of PASMCs increased TBK1/IKKε and YAP/TAZ activation

**FIGURE 2:**
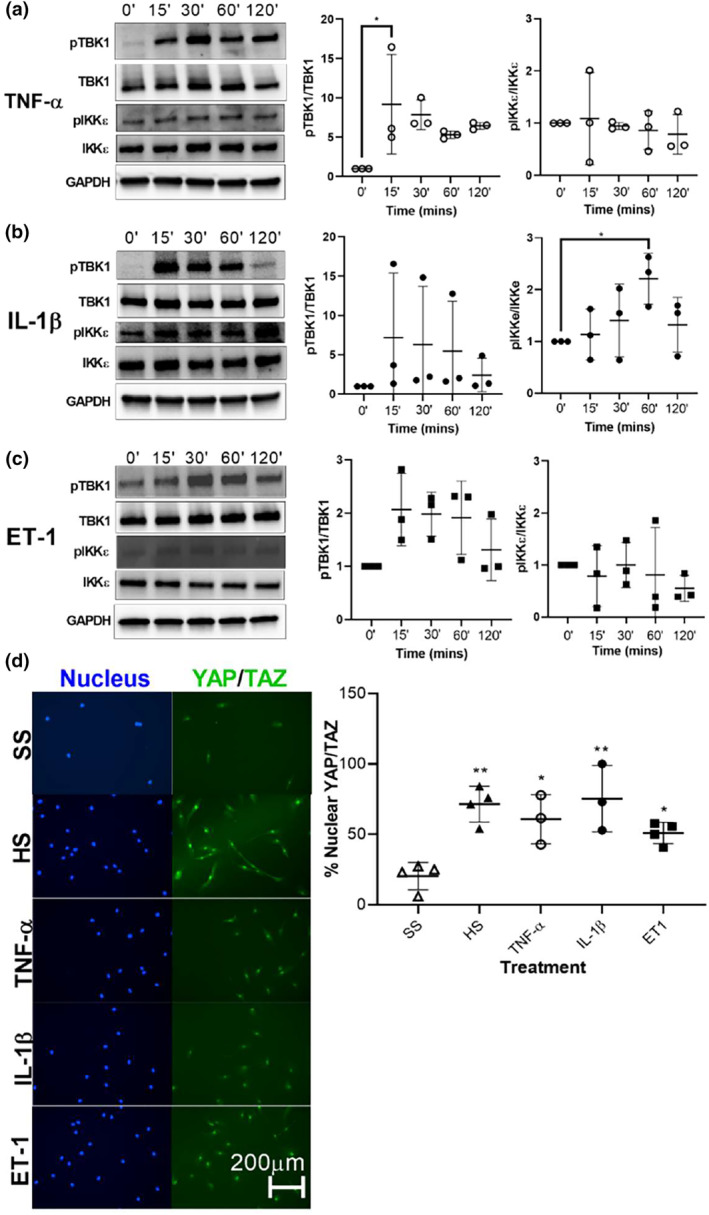
Effect of cytokine stimulation on PASMC TBK1/IKKε activation. PASMCs were stimulated with cytokines associated with PAH including (a) TNF‐α (20 ng/mL), (b) IL‐1β (10 ng/mL), or (c) ET‐1 (100 ng/mL). Total and phosphorylated TBK1, IKKε, and total GAPDH were quantified by densitometry. (d) PASMCs were serum starved or stimulated with high serum‐HS (20.0% FBS) media or cytokines TNF‐α, IL‐1β, or ET‐1 as above. Nuclear (active) YAP/TAZ in PASMCs was quantified by counting immunopositive. One‐way ANOVA with Tukey post‐test was used for both analyses, with significance designated * *p* < 0.05, ** *p* < 0.01. Horizontal lines indicate mean values. Data represent 3–4 biological experiments. Scalebar represents 200 μm.

Serum levels of cytokines such as TNF‐α and IL‐1β are increased in the serum of patients with PAH, and higher serum inflammatory cytokines are associated with poor survival in PAH over time (Cracowski et al., 2014; Soon et al., 2010). TNF‐α overexpression has been shown to increase lung volumes and induce PH in mice (Fujita et al., 2001; Stevens et al., 1992). TNF‐α treatment suppresses prostacyclin expression in rat PASMC (Itoh et al., 2003) and increases pulmonary vascular reactivity in isolated rat lungs (Stevens et al., 1992). Additionally, inhibition of TNF‐α and IL‐1β have been shown to attenuate MCT‐induced PH in rats (Voelkel et al., 1994; Wang et al., 2013). Endothelin‐1 (ET‐1) is a mediator of vasoconstriction in PAH and can also induce hyperproliferation of cells of the pulmonary vasculature, a hallmark of PAH. Given increased activation of non‐canonical IKB kinases in the MCT model of PH, we next assessed the effects of cytokine stimulation on activation of non‐canonical IKB kinases in PASMCs. Human PASMCs were stimulated with TNF‐α, IL‐1β, or ET‐1 and activation of TBK1 and IKKε (phosphorylated levels relative to total levels) were assessed at serial time points over 2 h. TNF‐α significantly increased TBK1 activation (Figure 2a), while IL‐1β significantly increased activation of IKKε (Figure 2b). TBK1 had a modest response to ET‐1 that failed to reach statistical significance (Figure 2c). Given the role of YAP/TAZ in PASMC activation (Dieffenbach et al., 2017) and given that TBK1 regulates YAP/TAZ activation in lung fibroblasts (Aravamudhan et al., 2020), we next examined the effect of these cytokines on YAP/TAZ activity in PASMCs. Treatment with TNF‐α, IL‐1β, and ET‐1 all increased nuclear (active) YAP/TAZ expression (Figure 2d) in serum‐starved PASMCs, similar to the high serum (HS) positive control. These results show that cytokines implicated in PAH activate non‐canonical IKB kinases and YAP/TAZ in human PASMCs.

### 3.3 Reducing TBK1/IKKε levels/activity in PASMCs decreases YAP/TAZ activation

**FIGURE 3:**
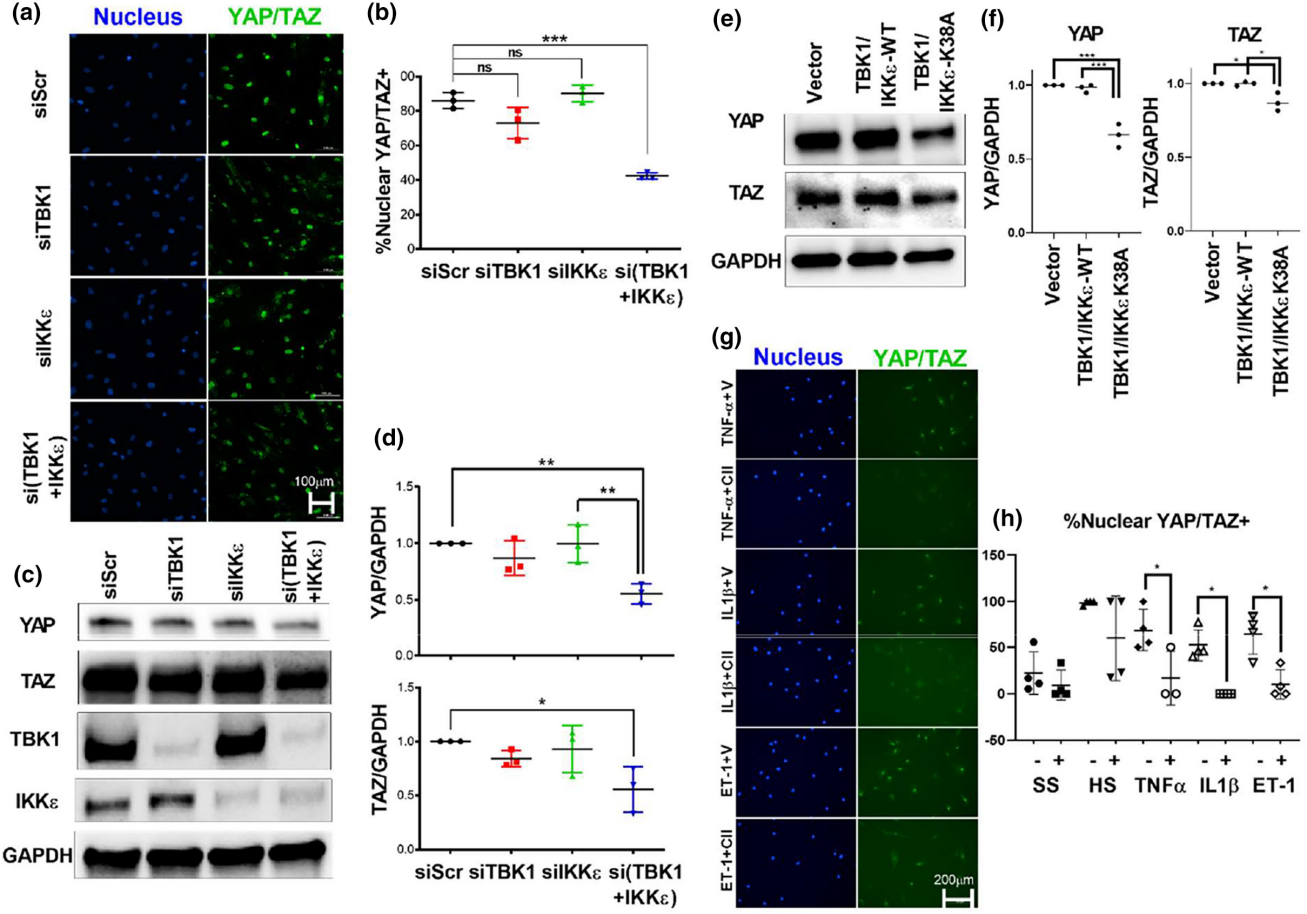
Reducing TBK1/IKKε decreases YAP/TAZ activation. (a) and (b): siRNA against TBK1 or IKKε alone did not reduce nuclear YAP/TAZ, but combined siRNA against both TBK1 and IKKε reduced nuclear YAP/TAZ significantly in PASMCs. (c) and (d) reducing TBK1 and IKKε on PASMCs together, significantly reduced the total protein levels of YAP/TAZ. (e) and (f): PASMCs transfected with kinase deficient TBK1K38A and IKKεK38A mutants had significantly lower levels of YAP and TAZ than wildtype (WT) and empty vector transfected cells. (g) and (h) Cytokine stimulation nuclear YAP/TAZ in PASMCs with TNF‐α, IL‐1β, and ET‐1 was reduced to baseline with a small molecule inhibitor (Compound II, +) of TBK1/IKKε compared to vehicle control (V, −) in each of the treatments. One‐way ANOVA with Tukey post‐test: Significance designated *p < 0.05, **p < 0.01, and ***p < 0.001. Horizontal lines indicate mean values. Data represent three or more biological experiments. Scalebar represents 100 or 200 μm as indicated.

To examine if biological silencing and chemical inhibition of TBK1/IKKε reduces activation of YAP/TAZ in PASMCs, siRNA, plasmid vector, and chemical inhibitors targeting TBK1/IKKε were administered to PASMCs (Figure 3). SiRNA against control (si‐Scr), TBK1 (si‐TBK1), IKKε (si‐IKKε), and a combination of TBK1/IKKε (si‐TBK1+IKKε) were administered to PASMCs. Nuclear YAP/TAZ levels were determined by imaging after immunostaining (Figure 3a), and the relative nuclear YAP/TAZ was quantified (Figure 3b). Combined knockdown of TBK1/IKKε (si‐TBK1+IKKε), but not individual knockdown, significantly decreased YAP/TAZ nuclear levels in PASMCs. To examine the effect of reducing TBK1 and IKKε on total protein levels of YAP/TAZ, PASMCs were treated with scramble control (si‐Scr), TBK1 (si‐TBK1), IKKε (si‐IKKε), and a combination of TBK1/IKKε (si‐TBK1+IKKε). Total protein was extracted and examined by western blot analysis (Figure 3c). Total protein levels of YAP and TAZ were significantly reduced in the si‐TBK1+IKKε group compared with si‐Scr control cells (Figure 3d). Like the effects on YAP/TAZ nuclear levels, individual silencing of TBK1 or IKKε alone did not significantly impact YAP or TAZ expression in PASMCs. These results show that silencing non‐canonical IKB kinase expression significantly reduces total and active (nuclear) YAP/TAZ levels in human PASMCs.

To determine if directly targeting the activity of non‐canonical IKB kinases affects YAP/TAZ expression, a plasmid containing vector control (Vector), wild type TBK1 and IKKε (TBK1/IKKε‐WT), and kinase dead mutants TBK1K38A and IKKεK38A (TBK1/IKKε‐K38A) were transfected into PASMCs. Western blot was run for YAP and TAZ (Figure 3e). Cells transfected with kinase dead mutants TBK1/IKKε‐K38A showed significantly decreased expression of YAP and TAZ compared with TBK1/IKKε‐WT‐transfected and control (vector) PASMCs (Figure 3f). These results suggest that the catalytic activity of TBK1/IKKε may be necessary to actively maintain protein levels of YAP/TAZ in PASMCs.

To further assess the effect of targeting non‐canonical IKB kinases chemically on YAP/TAZ nuclear localization, we treated cytokine‐stimulated PASMCs with the non‐canonical IKB kinase inhibitor Compound II (+) compared with vehicle (−) (Figure 3g). Cells were imaged after immunostaining for nuclear YAP/TAZ which demonstrated that CII treatment reversed cytokine‐stimulated activation of YAP/TAZ in PASMCs (Figure 3h). This further suggests that reducing the catalytic activity of non‐canonical IKB kinases attenuates YAP/TAZ activation in PASMCs.

### 3.4 TBK1/IKKε knockdown attenuates pathological vascular remodeling behaviors and YAP/TAZ function in PAH‐PASMC

**FIGURE 4:**
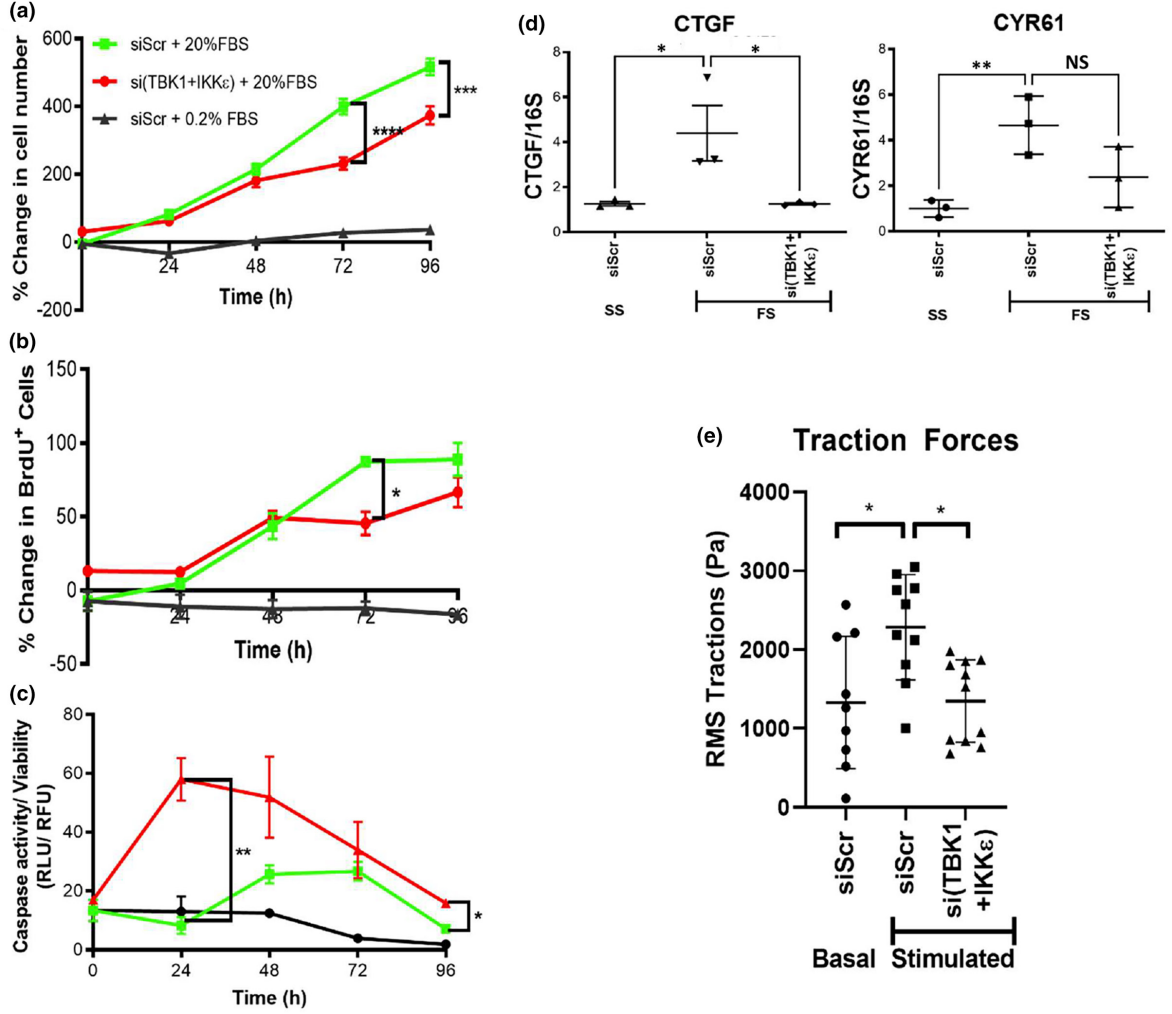
TBK1/IKKε knockdown reduces PASMC proliferation and contractile capacity. PASMC were grown in 20% FBS media transfected with siRNA scrambled control (si‐Scr) or targeting both TBK1 and IKKε (si‐(TBK1+IKKε)); as a comparison PASMC were grown in 0.2% FBS plus si‐Scr. Data in (a) and (b) are normalized to baseline controls collected at the time of siRNA treatment ~16 h prior to FBS stimulation. The PASMCs transfected with TBK1/IKKε showed (a) a net reduction in cell number at 72 h and 96 h, (b) lower proliferation at 72 h, and (c) increased apoptosis at 24 h. PASMCs transfected with si‐TBK1/IKKε also showed lower gene expression of (d) YAP/TAZ target genes CTGF and CYR61 in 20% FBS (FS), comparable to control levels seen in 0.2% FBS media (SS). (d) PASMCs increased root mean square (RMS) tractions when stimulated with TGF‐β (6 ng/mL) and ET‐1 (100 ng/mL) (Stimulated); this response was ablated by siRNA targeting TBK1 and IKKε. Tukey post‐test: Significance designated *p < 0.05, **p < 0.01, and ***p < 0.001. Horizontal lines indicate mean values. Data represent three or more biological experiments.

Pathologic hallmarks of PASMCs in PAH include excessive proliferation and resistance to apoptosis leading to an increase in cell number. We have also demonstrated enhanced contractility, assessed by traction force microscopy, in PAH PASMC (Dieffenbach et al., 2017). Our prior work showed that YAP/TAZ activity is increased in PAH PASMC and necessary for stiffness‐induced remodeling phenotypes. To establish the relationship between non‐canonical IKB kinases and PASMC pathological phenotype development, we knocked down TBK1/IKKε in PASMCs using siRNA (siTBK1+IKKε) and compared the effects to knockdown of a scramble control (siScr). PASMCs were then stimulated to proliferate in 20% FBS‐containing media, and a control group was maintained with 2% FBS‐containing media. Control samples were harvested and analyzed at the time of siRNA treatment to serve as a baseline. We assessed the effects of TBK1/IKKε knockdown on PASMC total cell number (Figure 4a), proliferation (by BrdU incorporation) (Figure 4b), and apoptosis (by caspase activity/viability) (Figure 4c). Knockdown of TBK1/IKKε significantly decreased proliferation (Figure 4b) and increased apoptosis (Figure 4c) with an overall reduction in PASMC cell number compared with controls at 72 h (Figure 4a). Furthermore, knockdown of TBK1/IKKε significantly reduced transcript levels of YAP/TAZ target genes *CTGF* and *CYR61* in 20% FBS‐stimulated PASMC compared with controls (Figure 4d), consistent with our observations of reduced YAP/TAZ protein and nuclear localization under these conditions. Knockdown of TBK1/IKKε also significantly attenuated contractility, as assessed by traction microscopy, in TGF‐β/ET‐1‐stimulated PASMCs (Figure 4e). These results demonstrate that a targeted specific reduction of non‐canonical IKB kinase expression significantly diminishes multiple PAH‐associated vascular remodeling behaviors in PASMCs.

### 3.5 TBK1/IKKε chemical inhibitors reduce active YAP/TAZ, cell number, and YAP/TAZ target genes in PASMC

**FIGURE 5:**
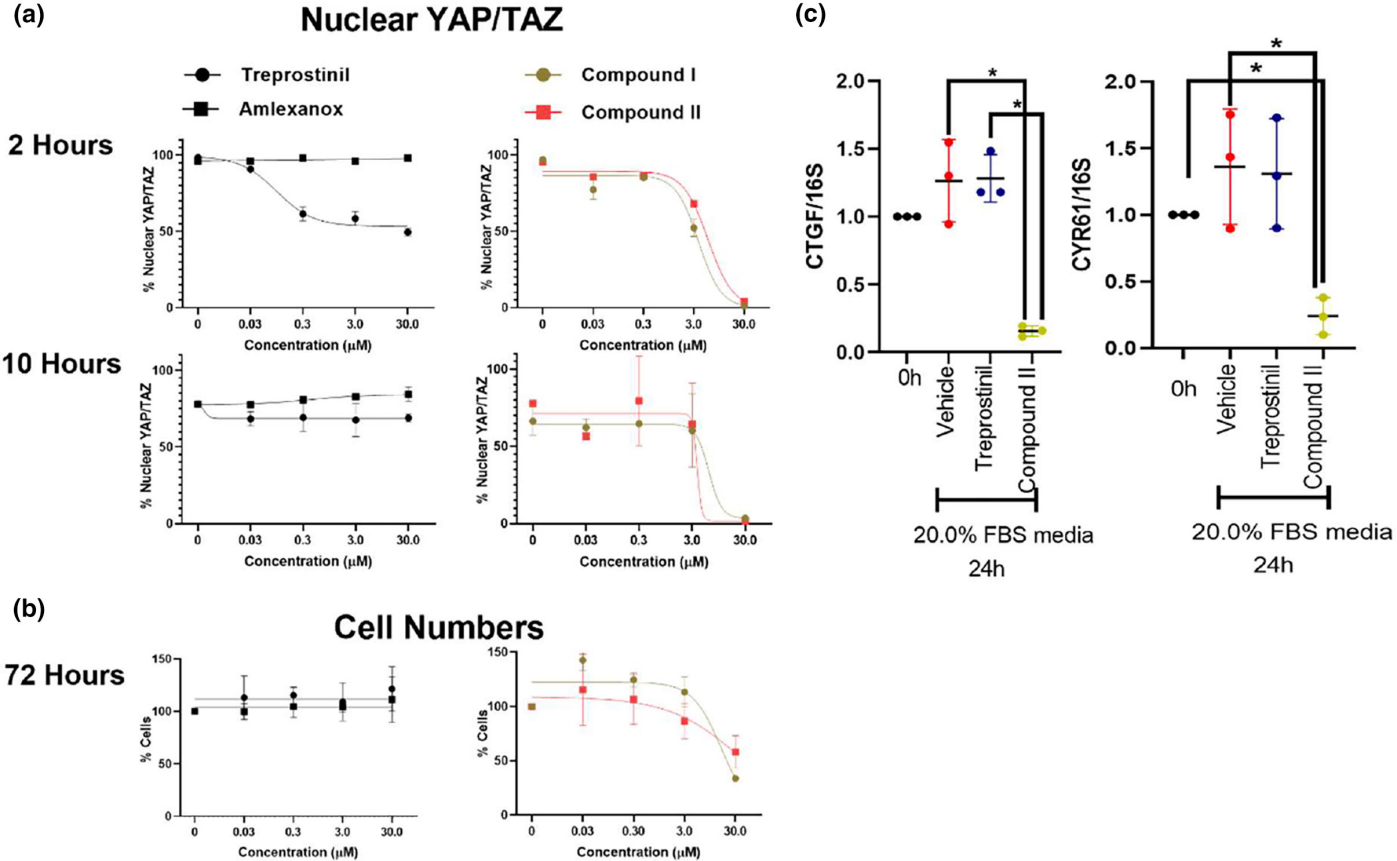
TBK1/IKKε inhibitors durably reduce nuclear YAP/TAZ, cell number, and YAP/TAZ target genes in PASMC. PASMC were stimulated with 20.0% FBS and treated with TBK1/IKKε inhibitors (amlexanox, Compound I, or Compound II), or the prostacyclin analog treprostinil (Trp). (a) YAP/TAZ nuclear levels were stably reduced in a dose‐dependent fashion at both 2 and 10 h after treatment with Compound I and Compound II. In contrast, amlexanox was ineffective, while treprostinil was potent but exerted only short‐duration effect at 2 h that was absent at 10 h. (b) Consistent with durable effects of Compounds I and II, cell numbers were also dose‐dependently reduced over 72 h of treatment, whereas no effect was seen with treprostinil. (c) Treatment of PASMC with Compound II durably reduced YAP/TAZ target genes CTGF and CYR61 levels 24 h after treatment, whereas treprostinil showed no effect at this time point. 5 μM of treprostinil, Compound II, or equivalent volume of DMSO (vehicle) were used in the experiment. One‐Way ANOVA and Tukey post‐test: Significance designated *p < 0.05. Horizontal lines indicate mean values. Data represent three or more biological experiments.

To test the effect of chemical inhibitors of non‐canonical IKB kinases on PAH‐associated vascular remodeling behaviors in PASMC, cells were stimulated with 20% FBS‐containing media. Treprostinil was used as an approved PAH therapeutic known to modulate YAP/TAZ via prostacyclin receptor activation (Liu et al., 2016; Zmajkovicova et al., 2019). Amlexanox (a non‐specific non‐canonical IKB kinase inhibitor), Compound I and Compound II (new generation of non‐canonical IKB kinase inhibitors) were administered over a concentration range of 0.03–30.0 μM. To determine the short‐ and long‐term effects of non‐canonical IKB kinase inhibitors on YAP/TAZ activity, nuclear (active) YAP/TAZ levels were assessed at 2 and 10 h after treatment (Figure 5a). Treprostinil reduced nuclear YAP/TAZ expression in a dose‐dependent manner at 2 h but was ineffective at 10 h. Amlexanox had no measurable effect, but both Compound I and Compound II showed dose‐dependent decreases in active YAP/TAZ at 2 h that were sustained out to 10 h. These results suggest that effective inhibition of non‐canonical IKB kinases can reduce YAP/TAZ activation for prolonged periods of time and may have more sustained effects on YAP/TAZ inactivation in PASMCs than treprostinil.

We also examined the impact of non‐canonical IKB kinase inhibitors on PASMC cell number at 72 h in 20% FBS‐containing media (Figure 5b). Neither amlexanox nor treprostinil had any effect on PASMC cell number at this extended duration. However, both Compound I and Compound II reduced PASMC numbers at 48 and 72 h, likely as one consequence of the prolonged YAP/TAZ inactivation observed with these non‐canonical IKB kinase inhibitors. Compound II was subsequently selected to be examined in comparison with treprostinil for its ability to modulate expression of YAP/TAZ downstream target genes. After 24 h of stimulation with 20% FBS‐containing media, cells treated with Compound II showed significantly lower expression of YAP/TAZ target genes CTGF and CYR61 compared with vehicle control and treprostinil‐treated PASMC groups (Figure 5c). These results highlight the potential of non‐canonical IKB kinase inhibitors to have a prolonged effect on proliferation and YAP/TAZ activity in PASMCs compared with prostacyclin analogs such as treprostinil.

## 4 DISCUSSION

The goal of this study was to determine if non‐canonical IKB kinases could be targeted to attenuate PAH‐associated pathological vascular remodeling behaviors and YAP/TAZ activation in PASMCs. Our previous work has demonstrated a key role for YAP/TAZ in driving vascular remodeling phenotypes in PASMCs (Dieffenbach et al., 2017). Our results show that TBK1/IKKε are activated in a rat model of PH and that cytokines implicated in PAH can activate TBK1/IKKε and YAP/TAZ in PASMCs. We also found that reducing the activity/levels of non‐canonical IKB kinases TBK1/IKKε decreased expression/activation of transcription factors YAP/TAZ in PASMCs. In addition to prolonged inactivation of YAP/TAZ, inhibition of TBK1/IKKε reduced proliferation, enhanced apoptosis, and decreased contractility in PASMCs, indicative of attenuating PAH‐associated vascular remodeling behaviors. These findings strongly suggest that the inhibition of non‐canonical IKB kinases offers a novel route to modulate YAP/TAZ in PASMCs in PAH.

System analysis of the human pulmonary arterial hypertension lung transcriptome previously showed an activation signature for several genes in the non‐canonical IKB kinase pathway including TBK1/IKKε, and interferon regulatory factors (IRF3/7) (Stearman et al., 2019). Several mediators and effectors of the non‐canonical IKB kinase pathway such as interleukin 1β (IL1β), Toll‐like receptors (TLRs), and interferon γ (IFNγ) were seen upregulated in a meta‐analysis of PAH blood genome (Elinoff et al., 2020). While most of our knowledge on non‐canonical IKB kinases comes from studies of innate immunity and cancer, the role of these kinases in a host of other pathophysiological signaling such as inflammation, cancer, diabetes, obesity, neurological disorders, and pulmonary fibrosis is rapidly emerging. Several diverse signaling events occur upstream of non‐canonical IKB kinase activation. Membrane‐bound TLRs, pattern recognition receptors (PRRs), or the cytosolic RNA and DNA sensors can activate TBK1/IKKε. Hence several cytokines, pathogen‐associated molecular patterns (PAMPS), and damage‐associated molecular patterns (DAMPS) can be upstream of these kinases and facilitate signaling in the non‐canonical IKB kinase pathway.

Of potential relevance to TBK1/IKKε activation in PAH, the initial oxidative stress/toxin‐induced endothelial injury of the pulmonary vasculature can lead to production of DAMPS (Kato et al., 2017; Mendonça et al., 2016). In addition, vasoconstriction that ensues from vascular remodeling in the endothelium may activate the inflammatory NF‐KB signaling pathway through TLR2 receptors (Li et al., 2013; Tan et al., 2014). A transcriptomic analysis of the MCT rat model of PH showed upregulation of DAMPs and an increase in TLR signaling (Xiao et al., 2020). PAMPS released by pathogens may also induce inflammation in PAH. Inflammation is a key characteristic feature of PAH and correlates with greater morbidity and mortality in PAH patients (Cracowski et al., 2014; Soon et al., 2010). Marked pulmonary perivascular inflammation correlates with intima and media remodeling in PAH and points to the role of inflammation in PAH‐associated vascular remodeling (Stacher et al., 2012). In animal models of PH (both MCT and Sugen/Hypoxia models), an inflammatory vascular response precedes the development of experimental PH (Meloche et al., 2017; Nogueira‐Ferreira et al., 2015). Furthermore, TNF‐α and IL‐1β are known to decrease prostacyclin synthesis and worsen disease in rat models of PH (Itoh et al., 2003). While the importance of inflammation in PAH is well established (Rabinovitch et al., 2014), the exact mechanism of inflammatory signaling in PAH pathogenesis remains unclear (Stacher et al., 2012). Our results support a novel potential link between inflammatory pathways and YAP/TAZ‐mediated PAH remodeling phenotypes via the non‐canonical IKB kinase pathway.

Several studies have also shown that YAP/TAZ may be activated in PAH via the altered mechanobiological environment of the remodeled vessels (Bertero et al., 2016; Dieffenbach et al., 2017; Liu et al., 2016). Moreover, reducing YAP/TAZ activation in the pulmonary vasculature ameliorates PH (Bertero et al., 2016; Zuo et al., 2021) in animal models. However, YAP/TAZ play critical roles in several cellular processes such as proliferation, differentiation, mechanoregulation, and development (Pocaterra et al., 2020). Hence, complete inhibition of YAP/TAZ in all cell types may be deleterious, even though drugs such as verteporfin and ROCK inhibitors could achieve that (Brouwer et al., 2021; Gibault et al., 2017; Noguchi et al., 2018). Understanding and targeting signaling events upstream of YAP/TAZ provides an opportunity to confine and target the effects of YAP/TAZ modulation to specific cell types/compartments of the cell avoiding deleterious side effects and maximizing efficacy in diseases driven by activation of YAP/TAZ, such as PAH. We show here that inhibition of non‐canonical IKB kinase pathway inhibits YAP/TAZ signaling downstream of multiple stimuli in PASMCs, highlighting its potential value.

Our prior work had shown the ability of non‐canonical IKB kinase TBK1 to regulate YAP/TAZ and the fibrogenic phenotype in pulmonary fibroblasts (Aravamudhan et al., 2020). IPF and IPAH share several common features and biological pathways. In a study comparing the pulmonary vascular gene expression profiles of patients with IPF and those who had consequent PH, no remarkable difference was found indicating that the pulmonary arteriolar changes in IPF and PH could have similar underlying mechanisms (Patel et al., 2013). However, another study comparing the lung gene expression profiles of patients with pulmonary fibrosis (PF) but no PH versus patients who had PF associated PH (APH) showed differential pathway regulation with the PF group being defined by a pro‐inflammatory signature and the APH group being more pro‐proliferative (Mura et al., 2012). Here, we showed that the link between non‐canonical IKB kinases and YAP/TAZ we observed in pulmonary fibroblasts extends to PASMCs, the matrix producing, contractile cells of the pulmonary vasculature implicated in PAH‐associated remodeling. Our results showed that TBK1/IKKε activity is essential to maintain the levels of active YAP/TAZ in PASMCs. Reducing TBK1/IKKε activity decreased active YAP/TAZ and attenuated PAH‐associated remodeling phenotypes for a sustained period compared with treprostinil, a currently approved PAH treatment, which was short‐acting. This finding points to a potential advantage in the durability of response in targeting PASMC YAP/TAZ activation via inhibition of non‐canonical IKB kinase pathway rather than agonism of the prostacyclin receptor.

Our study has several limitations. While our human cell studies confirmed TBK1/IKKԑ relevance in human cells from males and females, we analyzed in vivo TBK1/IKKԑ activation in only male rats, and future work should extend these observations to females. We used a limited number of primary human PASMCs and observed substantial donor‐to‐donor biological variability in some TBK1/IKKԑ responses. PAH is a heterogeneous disease and testing in larger populations of human cells will be needed to assess the broader utility of TBK1/IKKԑ inhibition in targeting PAH‐associated vascular remodeling behaviors. Similarly, testing of TBK1/IKKԑ inhibitors in pre‐clinical animal models of PAH will be needed to evaluate their safety and potential efficacy for PAH treatment.

In conclusion, our results demonstrate that reducing the activity or expression of TBK1/IKKε in PASMCs attenuates YAP/TAZ activation and vascular remodeling phenotypes. TBK1/IKKε are activated by inflammatory PAH‐associated cytokines and serve an essential role in YAP/TAZ protein stability by affecting YAP phosphorylation. Thus, we have identified non‐canonical IKB kinases TBK1/IKKε as important regulators linking cytokine‐based activation of YAP/TAZ in PASMCs. A new generation of TBK1/IKKε inhibitors with greater specificity and less toxicity are being discovered (Niederberger et al., 2013), providing a multitude of options to target the pathway. These results indicate that TBK1/IKKε inhibitors may offer a novel therapeutic approach to reduce YAP/TAZ activation and attenuate PASMC activation and downstream vascular remodeling in PAH.

## FUNDING INFORMATION

This work was supported by National Heart, Lung, and Blood Institute Grants 5R01HL137366–04 (to L.E.F. and D.J.T.); K08 HL‐143197 (to P.B.D.); American Heart Association (AHA) Grant‐in‐Aid 17GRNT33660449 (to L.E.F.) and AHA Grant 20POST35210650 (to A.A.)., HL105355 (to P.A.L.), HL158018 (to P.A.L.), and HL153026 (to P.A.L.).

## CONFLICT OF INTEREST STATEMENT

No conflicts of interest, financial or otherwise, are declared by the authors.

## 5 ETHICS STATEMENT

All animal experiments were performed in compliance with the laws and guidelines as set forth by the Harvard Medical area standing committee on animals and the Lovelace Respiratory Research Institute Animal Care and Use Committee. Human tissue samples and cell lines were obtained from Pulmonary Hypertension Breakthrough Initiative (PHBI) and under protocol approved by the Partners Research Committee. Informed consent was obtained by the PHBI from the subjects or their legal guardian prior to enrolling in the study.
